# A Differential Pattern of Batokine Expression in Perivascular Adipose Tissue Depots From Mice

**DOI:** 10.3389/fphys.2021.714530

**Published:** 2021-08-04

**Authors:** Alberto Mestres-Arenas, Joan Villarroya, Marta Giralt, Francesc Villarroya, Marion Peyrou

**Affiliations:** ^1^Departament de Bioquímica i Biomedicina Molecular and Institut de Biomedicina, Universitat de Barcelona, Barcelona, Spain; ^2^Centro de Investigación Biomédica en Red “Fisiopatología de la Obesidad y Nutrición”, Madrid, Spain; ^3^Institut de Recerca Hospital Sant Joan de Déu, Barcelona, Spain

**Keywords:** brown adipose tissue, white adipose tissue, thoracic perivascular adipose tissue, abdominal perivascular adipose tissue, batokine

## Abstract

Depending on its anatomical placement, perivascular adipose tissue (PVAT) has been found to possess features more (e.g., aortic thoracic) or less (e.g., aortic abdominal) similar to brown/beige adipose tissue in mice, whereas PVAT surrounding the mesenteric arteries and the caudal part of abdominal aorta is similar to white fat. PVAT is thought to influence vascular function through the effects of adipose-secreted molecules on vessels. Brown adipose tissue was recently shown to play differential secretory role *via* secretion of the so-called batokines but the involvement of differential batokine production in PVAT brown/beige plasticity was unclear. The current study characterizes for the first time the expression of batokines at aortic thoracic PVAT (tPVAT) and aortic abdominal PVAT (aPVAT) in comparison with typical brown and white adipose depots, in basal and thermogenically activated conditions. We found that both PVAT depots increased their expression of genes encoding the batokines bone morphogenetic protein-8b (BMP8B), fibroblast growth factor-21 (FGF21), and kininogen-2 (KNG2) in response to cold, indicating that, under cold-induced thermogenic activation, both thoracic aorta and abdominal aorta would experience intense local exposure to these PVAT-secreted batokines. In contrast, the gene expression levels of growth/differentiation factor-15 and vascular endothelial growth factor-A were induced only in tPVAT. Under short-term high-fat diet-induced thermogenic activation, the thoracic aorta would be specifically exposed to a local increase in PVAT-originating BMP8B, FGF21, and KNG2. Our data support the notion that acquisition of a brown/beige phenotype in PVAT is associated with upregulation of batokines, mainly BMP8B, FGF21, and KNG2, that can differentially target the vascular system.

## Introduction

Two types of adipose tissues have been traditionally distinguished in mammals: white adipose tissues (WAT) and brown adipose tissues (BAT). WAT, which is composed mostly of white adipocytes containing unilocular fat vacuoles, is the main site of metabolic energy storage in the body. BAT, meanwhile, is composed of brown adipocytes that contain multilocular lipid droplets and large amounts of mitochondria harboring the uncoupling protein-1 (UCP1) that enables the tissue to mediate adaptive thermogenesis (Cannon and Nedergaard, 2004). WAT is largely present at subcutaneous and visceral sites in experimental rodent models as well as in humans. In contrast, BAT is present at specific depots in the interscapular, subscapular, cervical, and other regions of rodents, whereas in humans, it is mostly present in the interscapular region of neonates, and mainly subscapular depots of adults (Cinti, 2001; Nedergaard et al., 2007). In recent years, a remarkable white-to-brown plasticity has been recognized under circumstances of enhanced thermogenic stimulus (e.g., a cold environment): Anatomical sites typically containing WAT (mostly subcutaneous) may become enriched in brown adipocyte-like cells that are termed “beige” adipocytes. These beige adipocytes are thermogenic and contain UCP1, but they are derived from a different cell lineage than the “classical” brown adipocytes present at anatomically defined BAT depots (Giralt and Villarroya, 2013; Chouchani and Kajimura, 2019). Whereas hypertrophied WAT, as in obesity, is associated with multiple diseases ranging from type II diabetes and cardiovascular disease to cancer, high BAT activity is positively correlated with cardio-metabolic health (Becher et al., 2021).

In addition to the roles played by WAT and BAT in the energy balance (storage vs. expenditure, respectively), the two types of adipose tissue are now recognized as playing different secretory roles. That of WAT has been extensively studied since the initial discovery of leptin, and over a hundred of “white” adipokines have been identified (Giralt et al., 2016). However, it was only recently that BAT was shown to have a relevant secretory activity distinct from that of WAT (Villarroya et al., 2017a). The pattern of BAT-derived secreted factors, termed “batokines,” appears to mediate autocrine, paracrine, and even endocrine effects distinct from those mediated by the WAT secretome (Villarroya et al., 2017b, 2019).

Perivascular adipose tissue (PVAT) is generally defined as a layer of adipose tissue that surrounds large vessels, such as the aortic artery. In rodents, histological (massive presence of multilocular adipocytes) and molecular (expression of *Ucp1* and other genes) characterization of PVAT has indicated that the aortic thoracic PVAT (tPVAT) resembles BAT, whereas PVAT around the abdominal aorta (aPVAT) exhibits a mixture of multilocular *Ucp1*-expressing adipocytes together with typical unilocular white adipocytes (Fitzgibbons et al., 2011). In humans, tPVAT also contains brown adipocyte-like cells, while aPVAT resembles more typical WAT (Chatterjee et al., 2009). PET scan-based functional data and histology analyses indicate that PVAT at human thoracic aorta resembles BAT (Wei et al., 2015). Despite these findings under basal conditions, however, we know little about the plasticity of PVAT in response to thermogenic challenges known to elicit BAT activation and WAT browning. Li et al. (2017) reported that cold exposure of rats induced *Ucp1* expression in both tPVAT and aPVAT, as it does in interscapular BAT and inguinal WAT (iWAT). Consistently, metabolic activation of tPVAT was noted in rats treated with a β3-adrenergic agonist, similar to what occurs in interscapular BAT (Mirbolooki et al., 2011). Mice exposed to mild cold (16°C) appear to express *Ucp1* more intensely in tPVAT, like in BAT (Chang et al., 2012), and Reynés et al. (2019) reported that cold exposure in ferrets (an animal model whose adipose tissue features and plasticity resemble those humans more closely than do those of rats and mice) induces thermogenic activation and represses pro-inflammatory immune pathways in peri-aortic tPVAT.

Increased PVAT fat accumulation (which is reminiscent of enhanced acquisition of white adipocyte-like morphology) is associated with cardiovascular risk (Montani et al., 2004), while increased browning at PVAT has been proposed to have protective effects (Aldiss et al., 2017). It is unknown how common it is for obesity phenotypes maintaining a healthy state to specifically influence PVAT functions. In general, the importance of PVAT for vascular health has been hypothesized to be associated with its secretory properties and the effects of adipocyte-derived factors on adjacent endothelial cells. Thus, enhanced secretion of pro-inflammatory factors, which is typical of hypertrophied white adipocytes, is considered to occur in high-fat-containing PVAT depots under conditions, such as obesity, thereby contributing to vascular damage (Oikonomou and Antoniades, 2017). However, we know little about how the brown/beige phenotype of PVAT at distinct anatomical sites and under distinct environmental conditions can help determine the pattern of batokine secretion and downstream influences on vascular functions.

Some batokines, such as kininogen-2 (KNG2) and possibly fibroblast growth factor-21 (FGF21), have been shown to influence the vascular system in experimental settings unrelated to a brown/beige adipose origin (Domouzoglou et al., 2015; Hamid et al., 2020). Vascular endothelial growth factor-A (VEGFA) and bone morphogenetic protein-8b (BMP8B) secreted specifically from brown adipocytes were shown to promote vascularization at BAT depots (Tonello et al., 1999; Xue et al., 2009; Pellegrinelli et al., 2018). However, the local secretory capacity of brown/beige-like PVAT depots has not been analyzed to date. The objective of our study was to characterize for the first time the expression of batokines at two distinct PVAT sites, aortic tPVAT and aPVAT, in comparison with typical BAT and WAT depots, under basal conditions and conditions of cold-induced and diet-induced thermogenic activation.

## Materials and Methods

### Animal Procedures

**Figure 1:**
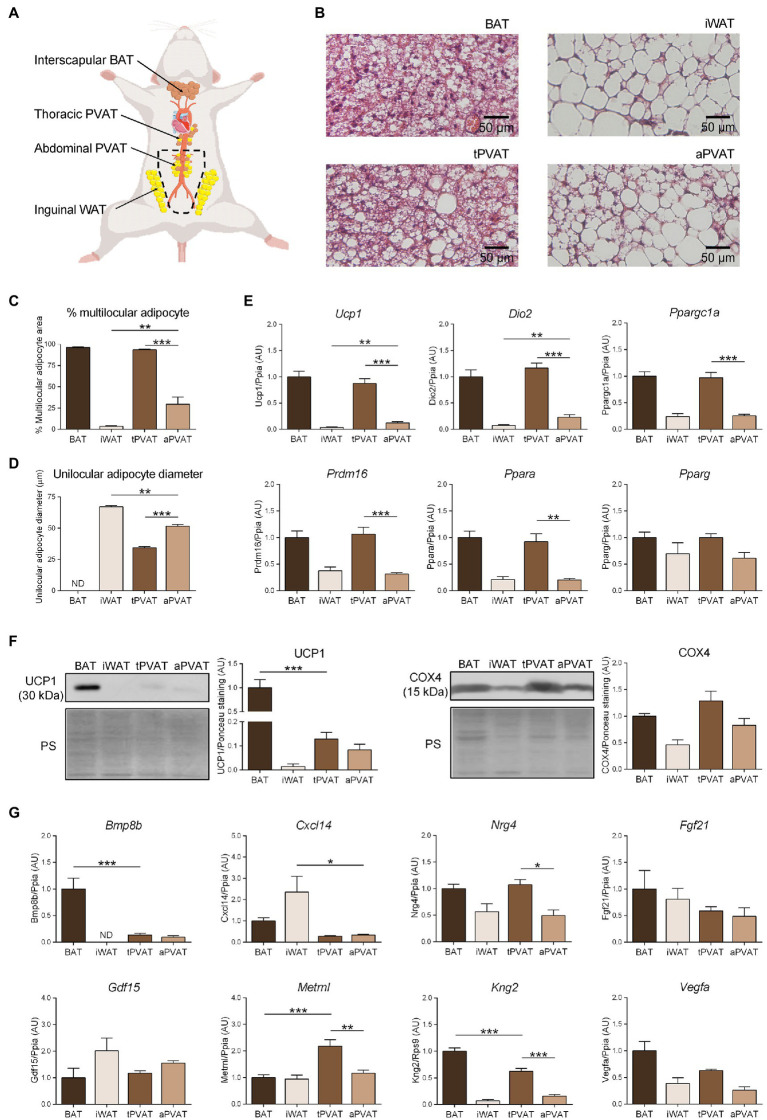
Characterization of perivascular adipose depots in mice under basal conditions. **(A)** Schematic representation of the anatomical placement of interscapular brown adipose tissues (BAT), inguinal WAT (iWAT), thoracic PVAT (tPVAT), and abdominal PVAT (aPVAT) depots in mice. **(B)** Representative optical microscopic pictures of hematoxylin-eosin stained sections of adipose tissue samples. **(C, D)** Quantitative assessment of cell morphology in adipose tissue depots. **(E)** Relative transcript levels of genes encoding components of the thermogenic and adipogenic machinery. **(F)** Relative levels of uncoupling protein-1 (UCP1) and cytochrome oxidase subunit IV (COX4) proteins (left, representative immunoblots; right, quantitative measurements). **(G)** Relative transcript levels of batokine-encoding genes. Bars correspond to means ± standard error of the mean (SEM) values from 4 to 6 mice. Statistical differences (^*^p < 0.05, ^**^p < 0.01, and ^***^p < 0.001) are shown for comparisons of BAT vs. tPVAT, iWAT vs. aPVAT, and tPVAT vs. aPVAT depot pairs. ND, non-detectable.

Experiments were conducted on 9- to 11-week-old male wild-type FVB/NJ mice, which were maintained at room temperature (21°C) or exposed to cold (4°C) for 1 week. Cold-exposed animals were housed in pairs. Total body weight and food consumption were monitored daily. Mice were maintained on a standard rodent diet (10% Kcal fat; Teklad Diet, Envigo) or, where indicated, fed a high-fat diet (HFD) for 4 weeks (^#^TD.06415, 45% Kcal fat, Teklad Custom Diet, Envigo). Body weight and energy intake of FVB/NJ mice before and after the different experimental settings are available in Supplementary Table 1. For all three experimental groups (control, cold-exposed, and HFD-fed individuals), mice were maintained under controlled humidity and a 12-h light/dark cycle (8:00 am–8:00 pm). For sample collection, animals were sacrificed by decapitation. Interscapular BAT, iWAT, tPVAT, and aPVAT depots were dissected (see Figure 1A) and weighed, and a piece was shock-frozen in liquid nitrogen and stored at −80°C for future RNA and protein analyses. All experimental procedures performed were authorized by the Institutional Animal Care and Use Committee of the University of Barcelona.

### Histological Analysis

BAT, iWAT, tPVAT, and aPVAT samples were collected, immediately fixed for 24 h in 4% paraformaldehyde, and then preserved in 70% ethanol. For analysis, the samples were dehydrated, embedded in paraffin blocks, cut into 5-μm-thick sections, and stained with hematoxylin and eosin. The stained sections were visualized with an Olympus BX61 light microscope at 20X and 4X. The percentage of multilocular adipocyte area and the mean unilocular adipocyte diameter were measured from digitalized images using the Fiji/ImageJ software. Multilocular adipocyte cell limits were manually drawn to measure area, then divided by the total area to obtain the percentage of multilocular adipocytes. For unilocular adipocyte, size measurements were obtained by manual drawing after initial setting of image scale. Results are average of at least 20 representative measurements per image in each of the 8–10 independent sections analyzed.

### Total RNA Isolation

Total RNA was extracted from tissue homogenates using a column-affinity-based methodology. A NucleoSpin RNA kit was utilized for BAT and iWAT samples and a NuceloSpin RNA XS kit was used for both perivascular samples (all from Macherey-Nagel), following the supplier’s recommendations. Final RNA yield was quantified using a NanoDrop ND-100 spectrophotometer (NanoDrop Technologies).

### Reverse Transcription Quantitative Real-Time Polymerase Chain Reaction Analysis

Reverse transcription was performed with 500 ng of total RNA employing a High Capacity RNA-to-cDNA kit (Applied Biosystems) according to the manufacturer’s instructions. Platinum Quantitative PCR SuperMix-UDG with ROX reagent or SYBR Select Master Mix (both from Thermo Fisher Scientific) was used as the master mix in a final reaction volume of 20 μl that contained specific TaqMan or SYBR Green probes (see Supplementary Table 2) for each gene of interest. An ABI 7500 Real-Time PCR System (Thermo Fisher Scientific) was used to perform reverse transcription quantitative real-time polymerase chain reaction (RT-qPCR) amplification. The relative expression levels of target genes in all samples were normalized to those of the *Ppia* or *Rps9* housekeeping genes using the 2^−AACt^ method (Livak and Schmittgen, 2001). For more information regarding RT-qPCR protocol, see MIQE guidelines in Supplementary Table 3.

### Western Blotting

Tissue samples for protein analysis were homogenized in RIPA buffer containing 50 mM Tris-HCl (pH 7.4), 150 mM NaCl, 0.1 mM EDTA, 0.1 mM EGTA, 1% NP-40, 0.5% sodium deoxycholate, 0.1% sodium dodecyl sulfate, 2 mM sodium orthovanadate, 10 mM *β*-glycerophosphate, 5 mM sodium fluoride, 1% phenylmethylsulfonyl fluoride, and a protease inhibitor cocktail (cOmplete-Mini, Roche). The protein concentration of each extract was determined by the BCA method (Thermo Fisher Scientific). Total protein (20 μg/lane) was resolved by SDS-PAGE in homemade 12% acrylamide/bisacrylamide gels and transferred to PVDF membranes (GE Healthcare). The membranes were incubated with primary antibodies specific for UCP1 (ab10983, Abcam) or cytochrome oxidase subunit IV (COX4; A-21342, Molecular Probes) diluted 1:1,000 in 5% BSA, followed by an incubation with the corresponding secondary antibodies HRP-conjugated anti-rabbit IgG (sc-2004, Santa Cruz) or anti-mouse IgG (170–6,516, Bio-Rad) diluted 1:3,000 in 5% milk. Signals were detected by chemiluminescence after addition of the Immobilon Western HRP substrate (Millipore), and digitalized images were quantified using Fiji/ImageJ software relative to total protein Ponceau staining.

### Statistical Analysis

Data are expressed as mean ± standard error of the mean with respect to BAT under the control condition, which was adjusted to have a mean = 1. Exact number of replicates is given in each figure legend. Putative outliers were detected and removed prior to statistical analyses using the Grubbs’ test, and the Shapiro-Wilk test was applied to establish the normality of datasets. A statistically significant difference between two groups was assessed by two-tailed unpaired Student’s *t*-test. For datasets that did not follow a normal distribution (*Cxcl14* transcript in basal conditions), differences were evaluated using a two-tailed unpaired nonparametric Mann-Whitney’s *U*-test. One-way ANOVA followed by Tukey Kramer’s multiple comparisons *post-hoc* test was used to compare three or more groups. Statistical analyses were performed with GraphPad Prism 8 (GraphPad Software Inc.) and the programming environment R. In all cases, the statistical significance threshold was set at *p <* 0.05.

## Results

Figure 1B shows the microscopic morphology of tPVAT and aPVAT in comparison with interscapular BAT (an adipose depot representative of classical BAT) and iWAT (as characteristic of subcutaneous WAT, a depot highly susceptible to browning). Under basal conditions, in which mice were maintained at 21°C and fed a standard diet, the microscopic morphology of tPVAT closely resembled that of BAT, with numerous multilocular adipocytes (more than 90%; Figure 1C). In contrast, the microscopic appearance of aPVAT was characterized by the presence of both unilocular and multilocular adipocytes. The percentage of multilocular adipocytes in aPVAT was much higher than in iWAT (Figure 1C) and the unilocular adipocytes were smaller in aPVAT than in iWAT (Figure 1D). Supplementary Figure 2 shows low magnification pictures of the adipose depots indicating the considerably homogeneous extent of morphological phenotype. The gene expression pattern reinforced the close similarity of tPVAT and BAT, as the whole set of transcripts for biomarkers of the brown/beige phenotype (i.e., *Ucp1*, *Dio2*, *Ppargc1a*, *Prdm16*, and *Ppara*) showed essentially equal expression levels in tPVAT relative to BAT (Figure 1E). Conversely, these transcripts were poorly expressed in aPVAT, where they were found in a range similar to that seen in iWAT, with the exception of a mild trend for higher expression of *Ucp1* in aPVAT. This observation may be due to less brown/beige adipocytes abundance in aPVAT. Meanwhile, the expression of *Pparg*, a gene commonly found in both brown/beige and white adipocyte phenotypes, was similar across all four adipose depots (Figure 1E).

We complemented this analysis by determining the protein levels of UCP1 and COX4, which is an indicator of the mitochondrial enrichment typical of brown/beige adipocytes. UCP1 protein was detected in tPVAT, but at a lower abundance than in BAT. In addition, while UCP1 protein level was very low in iWAT, aPVAT level appeared intermediate between PVAT and iWAT (Figure 1F). The differences were milder for COX4, but the highest levels were also found in BAT and tPVAT, and the lowest in iWAT and aPVAT (Figure 1F).

We next analyzed the expression patterns of batokine-encoding genes in the distinct adipose depots (Figure 1G). *Bmp8b* and *Kng2* exhibited an expression pattern resembling that of the brown/beige marker genes: Their expression levels were maximal in BAT, minimal in iWAT, relatively high in tPVAT (especially for *Kng2*), and low (but still above the minimal level in iWAT) in aPVAT. The expression levels of *Vegfa* and *Nrg4* followed an analogous but less intense pattern: They were higher in BAT and tPVAT and lower in iWAT and aPVAT (Figure 1G). On the other hand, *Cxcl14* expression followed a totally distinct pattern, with the highest expression found in iWAT and the lowest in both PVAT depots. *Metrnl* also followed a distinct expression profile, characterized by similar levels in the various adipose depots with the exception of tPVAT, in which *Metrnl* was significantly increased. Finally, no significant difference was found among the four adipose depots for *Fgf21* or *Gdf15* (Figure 1G).

**Figure 2:**
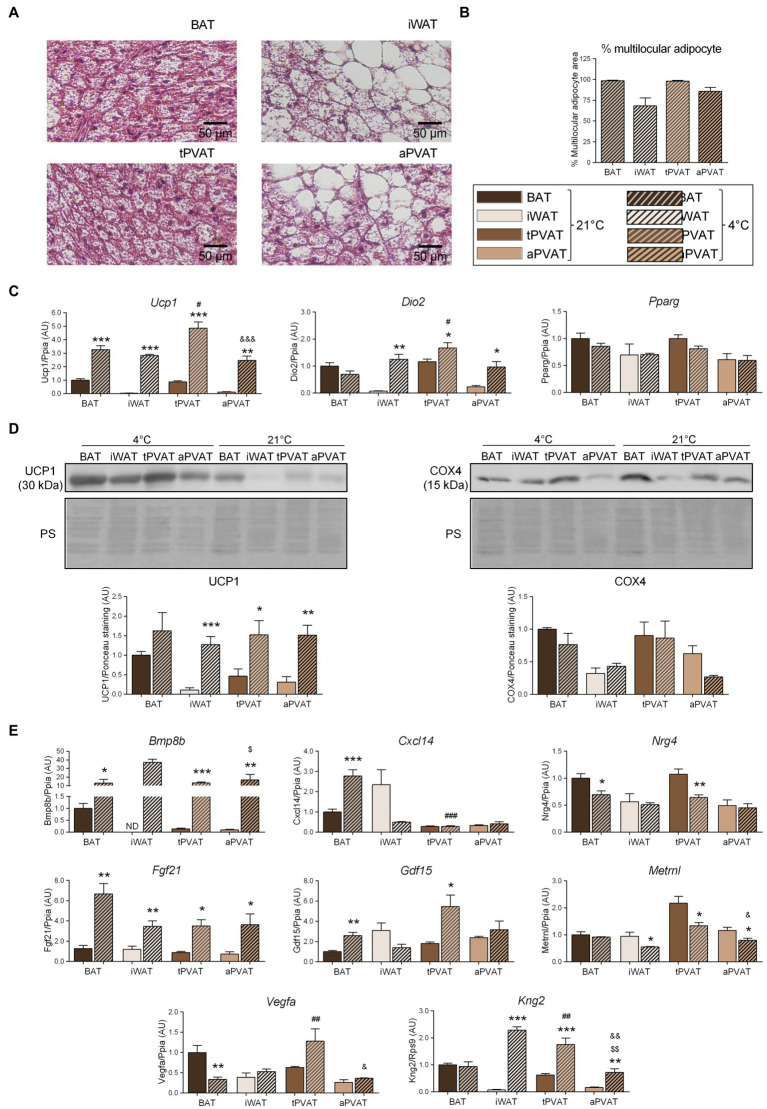
Effects of 1-week cold (4°C) exposure on perivascular adipose depots in mice. **(A)** Representative optical microscopic pictures of hematoxylin-eosin stained sections of adipose tissue samples. **(B)** Quantitative assessment of cell morphology in adipose tissue depots. **(C)** Relative transcript levels of genes encoding components FIGURE 2of the thermogenic and adipogenic machinery. **(D)** Relative levels of UCP1 and COX4 proteins (top, representative immunoblots; bottom, quantitative measurements). **(E)** Relative transcript levels of batokine-encoding genes. Bars correspond to means ± SEM values from 3 to 6 mice. Statistical differences (^*^p < 0.05, ^**^p < 0.01, and ^***^p < 0.001) are shown for the cold effect at each adipose depot. Comparison among depots in the cold condition is shown for BAT vs. tPVAT (^#^*p* < 0.05, ^##^*p* < 0.01, and ^###^*p* < 0.001), iWAT vs. aPVAT (^$^*p* < 0.05 and ^$$$^*p* < 0.001), and tPVAT vs. aPVAT (^&^*p* < 0.05, ^&&^*p* < 0.01, and ^&&&^*p* < 0.001). ND, non-detectable.

We then set out to determine the plasticity of mouse PVAT depots in response to a browning-inducing stimulus (exposure of mice to a cold environment for 1 week) and assess the extent to which such plasticity influences batokine expression. As expected, mice maintained at 4°C for 7 days exhibited a massive browning of iWAT, as assessed by cell morphology. Moreover, a large percentage of adipocytes acquired a multilocular morphology, which was not seen under the control situation at 21°C (Figure 2A). aPVAT experienced a similar but slightly more intense response to cold, with more than 75% of aPVAT cells acquiring a multilocular appearance after cold exposure (Figure 2B). Low magnification pictures (Supplementary Figure 2) indicated that, after cold, aPVAT shows a remarkable heterogeneity with areas showing predominant brown-like adipocytes, whereas in other areas, most cells remained with a white phenotype, rather similarly to what happens in iWAT. Cold-induced *Ucp1* mRNA expression was intense and fairly similar in tPVAT, aPVAT, BAT, and iWAT, with the highest cold-induced increase in *Ucp1* transcript levels seen in tPVAT (Figure 2C). A similar pattern of cold-induced expression was found at the distinct adipose depots for *Dio2*, another marker of brown/beige activation. The expression level of the general non-thermogenesis-related adipogenesis gene, *Pparg*, was unaltered at any of the adipose depots in response to cold. Parallel cold-induced increases were seen in the relative UCP1 protein levels at the four adipose depots, confirming our transcript-level findings, whereas COX4 protein levels were insensitive to cold-induced stimulation (Figure 2D).

Regarding the expression of batokine-encoding genes, the transcript levels of *Bmp8b*, *Cxcl14*, *Fgf21*, and *Gdf15* were increased in BAT in response to 1-week cold exposure (Figure 2E), in accordance with the previous reports (Hondares et al., 2011; Whittle et al., 2012; Cereijo et al., 2018; Campderrós et al., 2019). We found that 1 week of cold significantly reduced *Vegfa* mRNA in BAT, which is consistent with the previous data reporting that short-term, but not long-term, cold exposures induce *Vegfa* gene expression in BAT (Asano et al., 1997). Enhanced expression levels of *Bmp8b*, *Fgf21*, and *Kng2* were found in iWAT in association with cold-induced browning, also as previously reported (Fisher et al., 2012; Peyrou et al., 2020). The pattern of cold-induced expression changes in batokine-encoding genes were distinct according to the type of PVAT: *Bmp8b*, *Fgf21*, and *Kng2* were strongly induced in response to cold both in tPVAT and aPVAT, whereas *Gdf15* was only induced in tPVAT (as also seen in BAT). The *Cxcl14* transcript was not altered at any site other than BAT, while the expression levels of *Nrg4*, *Vegfa*, and *Metrnl* mRNAs were not induced by cold. Indeed, the opposite was observed as: *Nrg4* expression was downregulated by cold in both BAT and tPVAT, *Vegfa* was decreased in BAT, and *Metrnl* expression was reduced in all adipose depots tested, except for BAT.

**Figure 3:**
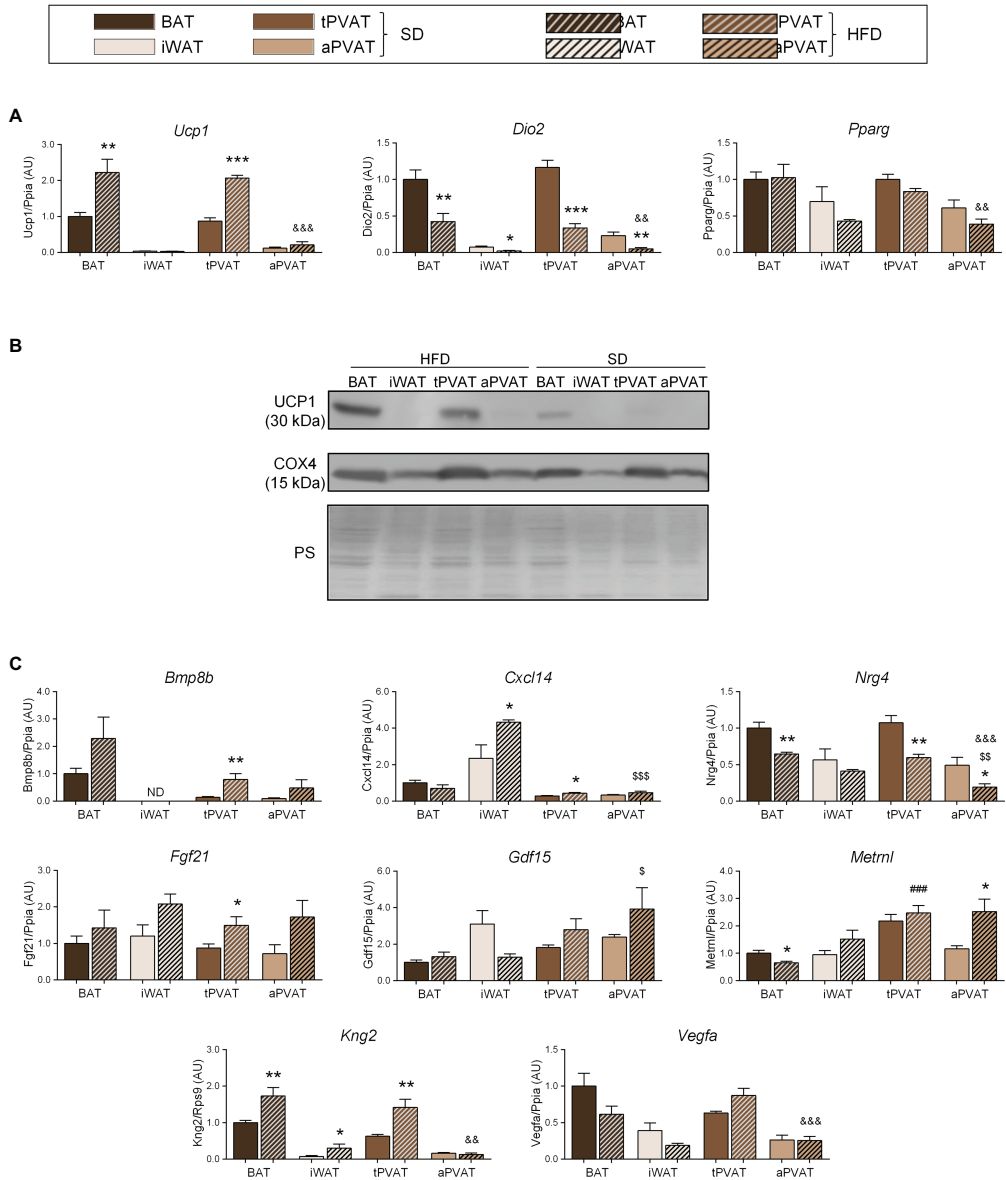
Effect of HFD feeding for 1 month on perivascular adipose depots in mice. **(A)** Relative transcript levels of genes encoding components of the thermogenic and adipogenic machinery. **(B)** Representative immunoblots of UCP1 and COX4 proteins. **(C)** Relative transcript levels of batokine-encoding genes. Bars correspond to means ± SEM from 5 to 6 mice. Significant differences (^*^p < 0.05, ^**^p < 0.01, and ^***^p < 0.001) are shown for HFD feeding effect at each adipose depot. Comparison among depots in the HFD-fed condition is shown for BAT vs. tPVAT (^###^*p* < 0.001), iWAT vs. aPVAT (^$$^*p* < 0.01 and ^$$$^*p* < 0.001), and tPVAT vs. aPVAT (^&&^*p* < 0.01 and ^&&&^*p* < 0.001). ND, non-detectable.

We next expanded our study to a second distinct inducer of adipose tissue plasticity. For this purpose, we treated mice with a HFD for 1 month, which was reported to represent a relatively short experimental design favoring diet-induced thermogenesis and UCP1 induction at BAT (Fromme and Klingenspor, 2011). Consistent with these reports, HFD upregulated *Ucp1* transcript (Figure 3A) and UCP1 protein (Figure 3B) levels in BAT but not in iWAT. Notably, an identical pattern of changes was observed for tPVAT relative to BAT, while aPVAT behaved like iWAT. Expression of *Dio2*, which is an indicator of cold-induced thermogenesis in BAT and iWAT, was downregulated in response to HFD in all four depots (Figure 3A), whereas HFD feeding did not cause remarkable changes in *Pparg* expression in any of the analyzed adipose tissues.

The transcript expression levels of the batokines *Bmp8b* and *Fgf21* trended higher in response to HFD at all adipose depots, except for *Bmp8b* expression in iWAT, which was undetectable. Whereas changes due to HFD were statistically significant only at tPVAT, ANOVA of the whole set of adipose depots revealed that the “diet factor” is associated with overall significant increases in the transcript levels of *Bmp8b* (*p* = 0.0092) and *Fgf21* (*p* = 0.0017). Likewise, a significant HFD-induced increase in BAT and tPVAT, but not in aPVAT, was found for *Kng2*. *Cxcl14* expression was induced by HFD in iWAT and tPVAT, whereas *Metrnl* was induced by HFD only in aPVAT. *Nrg4* expression was down-regulated in response to HFD at all of the analyzed adipose depots, although not to a significant degree in iWAT, whereas *Vegfa* expression was downregulated by HFD in BAT and iWAT, but not in either PVAT (Figure 3C).

## Discussion

Microscopy morphology (percentage of multilocular adipocytes), gene expression, and relative protein abundance of brown/beige phenotype markers under basal conditions indicated that there was a strong resemblance between tPVAT and classical BAT, and also a close similarity between aPVAT and iWAT. This is in agreement with the previous reports in mice and other rodent models (Fitzgibbons et al., 2011; Chang et al., 2012; Reynés et al., 2019). Further, we found that tPVAT responded to cold-induced thermogenesis by eliciting a pattern of thermogenesis-related gene expression similar to that observed in BAT, whereas aPVAT resembled iWAT in that it showed a strong capacity for browning in response to cold stimulus. These findings indicate a strong plasticity capacity of mouse PVAT to acquire a phenotype of activated BAT (tPVAT) and beige (aPVAT) adipose tissues in response to cold and thus confirm the previous reports in rodents (Chang et al., 2012; Li et al., 2017) and in other animal models (Reynés et al., 2019). Meanwhile, we showed that short-term feeding of a HFD induced signs of thermogenic activation (induction of *Ucp1* expression) in BAT and tPVAT, indicating that their resemblance is not limited to the cold response, but also concerns diet-induced thermogenic activation. We saw in our study that the extent of *Ucp1* mRNA expression and UCP1 protein levels at PVAT depots under distinct conditions is not always concordant. There are several reports indicating specific regulation of *Ucp1* mRNA translation to UCP1 protein as part of the thermogenic machinery of brown adipocytes and in response to thermogenic stimuli (Dai et al., 2015; Chen et al., 2018). Thus, it cannot be ruled out that translation efficiency of *Ucp1* transcripts was different in BAT relative to PVAT depots.

Under basal conditions, several batokines (*Bmp8b*, *Nrg4*, *Kng2*, and *Vegfa*) exhibited mRNA expression patterns in tPVAT and aPVAT that were similar to those in BAT and iWAT, respectively. This suggests that, at the most common environmental temperature conditions for rodent models (21°C), the thoracic section of the aortic artery is highly exposed to the local secretion of these factors by PVAT, whereas the abdominal aorta has much less such exposure. Additionally, our findings suggest that PVAT shows a highly sensitive ability to modify its batokine expression profile, following a distinct pattern according to the PVAT depot and for each specific batokine. For example, tPVAT and aPVAT increased their expression levels of *Bmp8b*, *Fgf21*, and *Kng2* in response to cold, whereas *Gdf15* and *Vegfa* were induced in tPVAT but not in aPVAT. Nevertheless, there is a remarkable capacity of aPVAT, which possesses low-level expression of most batokines under basal condition, to induce the expression of *Bmp8b*, *Fgf21*, and *Kng2* transcripts in response to cold. In response to a short-term HFD, a milder thermogenic stimulus, the induction of batokine gene expression was more restricted to tPVAT, and basically concerned the transcripts for *Bmp8b*, *Fgf21*, and *Kng2*. Based on our findings, we hypothesized that under cold conditions, both the thoracic and the abdominal aorta experience intense local exposure to these PVAT-synthesized and secreted batokines. Under short-term HFD, in contrast, only the thoracic aorta is exposed to local increases in PVAT-originating BMP8B, FGF21, and KNG2.

Several indirect lines of evidence suggest that batokines expressed in BAT/beige-activated PVAT may exert relevant effects on vascular function. In this context, classical BAT-secreted BMP8B has been shown to promote vascularization and induce the expression of angiogenic factors (Pellegrinelli et al., 2018). Thus, we can expect that local secretion of BMP8B by brown/beige-activated PVAT could exert analogous effects on attached large vessels. Published findings suggest that kininogens, which are key components of the kinin-kallikrein system, play a role in the cardiovascular system, such as preventing hypertension and ischemia (Hamid et al., 2020). *Gdf15* and *Vegfa* appear to be particularly induced in tPVAT in response to cold. GDF15 has been reported to modulate vascular contraction and relaxation responses in an endothelium-dependent fashion (Mazagova et al., 2013), whereas VEGFA is a very well-known angiogenic factor. Overall, these findings indicate that the acquisition of an active BAT-like phenotype and browning features in PVAT depots may result in a distinct pattern of batokine secretion, implying a differential functional impact on vascular cells distinct from the standard adipokine secretion elicited from conventional white fat. Thus, it is tempting to speculate that enhanced PVAT batokine secretion would play a cardiovascular-protective role, especially since Chang et al. (2012) showed that mild cold-induced PVAT activation attenuates age-dependent and obesity-induced endothelial dysfunction in mice. However, the fact that our study is based on transcriptomic data is a limitation, and further research is needed to confirm regulated production of batokines by PVAT depots as suggested by transcript-based data.

We know far less about human PVAT compared to that in rodent models and there are indications that human PVAT has somewhat distinct features relative to mice. In spite of that, recent studies reported that human tPVAT shows some BAT/beige features (for instance *Ucp1* expression; Wei et al., 2015; Vargas et al., 2018; Hamid et al., 2020), whereas aPVAT resembles WAT. The plasticity of human PVAT depots in response to thermogenic stimuli is unknown, but a recent study based on autopsies of Siberian adults indicated that almost one half the individuals assessed exhibited multilocular adipocytes, paucilocular adipocytes, and *UCP1* expression in mediastinal peri-aortic PVAT (Efremova et al., 2020).

## Conclusion

We herein report that PVAT batokine expression is generally concordant with the distinct resemblances of tPVAT and aPVAT to BAT and iWAT, respectively. Moreover, there is a remarkable plasticity of PVAT depots to acquire features of activated BAT (tPVAT) and beige (aPVAT) adipose tissues in response to thermogenic stimuli, and this is paralleled by a distinct expression pattern of batokines. Further research is warranted to determine how the individual batokines released by PVAT depots of distinct BAT/beige phenotypes specifically impact adjacent vascular structures at upper and lower sites of main vessels.

## Data Availability Statement

The raw data supporting the conclusions of this article will be made available by the authors, without undue reservation.

## Ethics Statement

The animal study was reviewed and approved by Institutional Animal Care and Use Committee of the University of Barcelona (approval code 9292).

## Author Contributions

MP, MG, and FV designed the study. AM-A and JV obtained the samples. AM-A performed the analytical procedures. MP and FV wrote the manuscript. All authors discussed the data and approved the final version.

## Conflict of Interest

The authors declare that the research was conducted in the absence of any commercial or financial relationships that could be construed as a potential conflict of interest.

## Publisher’s Note

All claims expressed in this article are solely those of the authors and do not necessarily represent those of their affiliated organizations, or those of the publisher, the editors and the reviewers. Any product that may be evaluated in this article, or claim that may be made by its manufacturer, is not guaranteed or endorsed by the publisher.
