# Supplementary material for: A Differential Pattern of Batokine Expression in Perivascular Adipose Tissue Depots From Mice
Source: Front Physiol. 2021 Aug 4;12:714530. doi: 10.3389/fphys.2021.714530 (PMC8373243; doi:10.3389/fphys.2021.714530)
Supplement: Supplementary file 1 [file Data_Sheet_1.docx]

***Supplementary material***

**A differential pattern of batokine expression in perivascular adipose tissue depots from mice**

**Alberto Mestres-Arenas et al.**

***Correspondence:** Dr. Marion Peyrou, peyrou.marion@gmail.com

**1 Supplementary Table 1:** Body weight and energy intake of FVB/NJ mice before and after the different experimental settings. Data are reported as mean ± standard deviation.

|  | **Body weight (g)** | | **Energy intake (kcal/day)** | |
| --- | --- | --- | --- | --- |
|  | **Beginning of experiment** | **Sacrifice (9-11 weeks old)** | **Beginning of experiment** | **Sacrifice (9-11 weeks old)** |
| **Basal** |  | 27.64 ± 2.06 |  | 12.11 ± 0.34 |
| **Cold (4°C 1 week)** | 27.08 ± 1.71 | 28.58 ± 1.30 | 13.09 ± 3.67 | 15.88 ± 6.17 |
| **HFD (4 weeks)** | 23.34 ± 1.01 | 33.95 ± 2.10 | 12.43 ± 0.35 | 16.98 ± 1.02 |

**2 Supplementary Table 2:** TaqMan assays and primers used for SYBRGreen analysis in qRT-PCR analysis of RNA transcript levels.

**a) TaqMan probes**

| **Primer name** | **Gene symbol** | **Catalogue number** | **Amplicon length (bp)** | **Location (exon boundary)** |
| --- | --- | --- | --- | --- |
| **Reference Gene** |  |  |  |  |
| Peptidylprolyl isomerase A | *Ppia* | Mm 02342430_gl | 148 | 4-5 |
| **Thermogenesis** |  |  |  |  |
| Uncoupling protein 1 | *Ucp1* | Mm 00494069_m1 | 95 | 1-2 |
| Iodothyronine deiodinase 2 | *Dio2* | Mm 00515664_m1 | 77 | 1-2 |
| Pparg coactivator 1 alpha | *Ppargc1a* | Mm 00447183_m1 | 104 | 5-6 |
| PR/SET domain 16 | *Prdm16* | Mm 00712556_m1 | 60 | 14-15 |
| Peroxisome proliferator activated receptor gamma | *Ppara* | Mm 00440939_m1 | 74 | 7-8 |
| Peroxisome proliferator activated receptor alpha | *Pparg* | Mm 00440945_m1 | 105 | 6-7 |
| **Batokines** |  |  |  |  |
| Bone morphogenetic protein 8b | *Bmp8b* | Mm 00432115_g1 | 77 | 6-7 |
| C-X-C motif chemokine ligand 14 | *Cxcl14* | Mm 00444699_m1 | 64 | 3-4 |
| Neuregulin 4 | *Nrg4* | Mm 00446254_m1 | 105 | 5-6 |
| Fibroblast growth factor 21 | *Fgf21* | Mm 00840165_g1 | 78 | 1-2 |
| Growth differentiation factor 15 | *Gdf15* | Mm 00442228_m1 | 72 | 1-2 |
| Meteorin like | *Metrnl* | Mm 00522681_m1 | 64 | 3-4 |
| Vascular endothelial growth factor A | *Vegfa* | Mm 01281449_m1 | 81 | 3-4, 4-5, 6-7, and 7-8 |

**b) Oligonucleotides used for SYBRGreen analysis**

| **Primer name** | **Gene symbol** | **Primer Sequence**  **5’ to 3’** | **Catalogue number** | **Amplicon length (bp)** | **Location (exon)** |
| --- | --- | --- | --- | --- | --- |
| **Reference Gene** |  |  |  |  |  |
| Ribosomal protein S9 | *Rps9* | F 5’ GACCAGGAGCTA  AAGTTGATTGGA 3’ | SY150138747-079 | 81 | exon 2 |
|  |  | R 5’ TCTTGGCCAGG  GTAAACTTGA 3’ | SY150138747-080 |  |  |
| **Batokines** |  |  |  |  |  |
| Kininogen 2, high molecular weight kininogen* | *Kng2 HMWK* | F 5’ GACTGCCCAGA  GAACAGAACC 3’ | SY150138747-073 | 73 | exon 9 |
|  |  | R 5’ CCTCTCCTGTA  TCTGTGTAGA 3’ | SY150138747-074 |  |  |
| Kininogen 2, low molecular weight kininogen* | *Kng2 LMWK* | F 5’ GCAGGAACAAC  TAGGCTCCTA 3’ | SY150227968-044 | 156 | exon 9 |
|  |  | R 5’ CATCTCAGGAT  TCTTCTGCTCC 3’ | SY150227968-045 |  |  |

* Kng2 high and low molecular weight isoforms were analyzed separately using specific probes. Given the similarity of the results obtained, data from both isoforms have been fused and plotted together in a single graph.

**3 Supplementary Table 3:** detailed information of the qRT-PCR analyses according to the MIQE guidelines.

| **Item to check** | **Provided** | **Comment** |
| --- | --- | --- |
| **1. Specimen** |  |  |
| Detailed description of specimen type and numbers | Y | 9-to 11-week-old male wild-type FVB/NJ mice maintained under controlled humidity and a 12-hour light/dark cycle (8:00am-8:00pm). Three different experimental conditions have been assessed: control animals (kept at room temperature of 21°C and a standard rodent diet containing 10% kcal fat, n=12); cold exposed mice (kept at 4°C during 1 week and fed the same standard diet, n=9); and high-fat diet-fed individuals (kept at room temperature of 21°C and fed a high-fat diet containing 45% kcal fat, n=6). |
| Sampling procedure (including time to storage) | Y | For sample collection, animals were sacrificed by decapitation. The different adipose depots analyzed (BAT, iWAT, tPVAT and aPVAT) were dissected, weighed, and immediately shock-frozen in liquid nitrogen. |
| Sample aliquotation, storage conditions and duration | Y | Tissue samples (aliquoted as approximately 30mg for BAT, 60mg for iWAT, and all the depot for tPVAT and aPVAT) were transferred from liquid nitrogen to a −80°C freezer within one hour and were kept there until processing (1-2 weeks). |
| **2. Nucleic acid extraction** |  |  |
| Description of extraction method including amount of sample processed | Y | Tissue fragments (around 30mg for BAT, 60mg for iWAT, and all the depot for tPVAT and aPVAT) were physically and chemically digested in an Eppendorf tube containing a metallic bead and 500μL of digestion solution (RA1+β-mercaptoethanol) for 90 seconds oscillating at 50rpm. Then, the RNA was extracted from tissue homogenates using a column-affinity based methodology. A NuceloSpin RNA kit was utilized for BAT and iWAT samples and a NucleoSpin RNA XS kit was used for both perivascular samples (all from Macherey-Nagel), following the supplier's protocol. |
| Volume of solvent used to elute/resuspend extract | Y | Extracted RNA was eluted in 40μL of RNase-free water for BAT and iWAT samples, and in 10μL for tPVAT and aPVAT samples. |
| Number of extraction replicates | N | There was no possibility to perform replicate extractions from perivascular adipose depots given that all the frozen sample was used in each of the extractions done. |
| Extraction blanks included? | N | Not done. |
| **3. Nucleic acid assessment and storage** |  |  |
| Method to evaluate quality of nucleic acids | Y | Protein and phenol contaminations were assessed on the basis of spectrophotometry (260nm, 230nm, 280nm) using a NanoDrop ND-100 spectrophotometer (NanoDrop Technologies). |
| Method to evaluate quantity of nucleic acids (including molecular weight and calculations when using mass) | Y | Final RNA yield was quantified using a NanoDrop ND-100 spectrophotometer (NanoDrop Technologies). |
| Storage conditions: temperature, concentration, duration, buffer, aliquots | Y | Eluted RNA was maintained in a −20°C freezer at its given concentration until further processed in a few days. |
| Clear description of dilution steps used to prepare working DNA solution | N | Not applicable to our experimental design. |
| **4. Nucleic acid modification** |  |  |
| Template modification (digestion, sonication, pre-amplification, bisulphite etc.) | N | Not applicable to our experimental design. |
| Details of repurification following modification if performed | N | Not applicable to our experimental design. |
| **5. Reverse transcription** |  |  |
| cDNA priming method and concentration | Y | cDNA priming was based on random octamers plus oligo-dT16 (contained in the Applied Biosystems kit, no concentration data available). |
| One or two step protocol (include reaction details for two step) | Y | RNA was converted into cDNA in a two-step manner. First, reverse transcription was carried out in a total volume of 20μL, and then 1μL of the resulting cDNA was added in the RT-PCR reaction mixture of also 20μL. |
| Amount of RNA added per reaction | Y | 500ng of total RNA in RNase-free water were used for reverse transcription in a partial volume of 9μL. |
| Detailed reaction components and conditions | Y | Reverse transcription was performed with 500ng of total RNA (in 9μL), with 10μL of RT buffer (containing dNTPs, random octamers, and oligo-dT16), and 1μL of enzyme mix (containing MuLV MultiScribeTM reverse transcriptase and RNase protein inhibitors). Reverse transcription of the resulting 20μL reaction mix was performed in a thermocycler at the following conditions: 25°C for 10 minutes, 48°C for 30 minutes, and 95°C for 5 minutes. |
| Estimated copies measured with and without addition of RT | Y | All RNA samples yielded > 40 CTs in qRT-PCR assays without RT. |
| Manufacturer of reagents used with catalogue and lot numbers | Y | Applied Biosystems (reference nº 4387406). |
| Storage of cDNA: temperature, concentration, duration, buffer and aliquots | Y | Final cDNA was maintained in a −20°C freezer at its given concentration until further processed. |
| **6. dPCR oligonucleotides design and target information** |  |  |
| Sequence accession number or official gene symbol | Y | Provided in Supplementary Table 2. |
| Method (software) used for design and in silico verification | Y | Not available from supplier for TaqMan probes; PearlPrimer software used for the design of SyberGreen primers. |
| Location of amplicon | Y | Provided in Supplementary Table 2. |
| Amplicon length | Y | Provided in Supplementary Table 2. |
| Primer and probe sequences (or amplicon context sequence) | Y | The use of TaqMan-bassed assays (Applied Biosystems) does not allow to disclose this information. References to access to the limited information by supplier are shown in Supplementary Table 2. Primer sequences for SYBRoligos are provided in Supplementary Table 2. |
| Location and identity of any modifications | N | Not applicable. |
| Manufacturer of oligonucleotides | Y | Applied Biosystems (for TaqMan assays) and Merck Life Sciences (for SYBRoligos). |
| **7. dPCR protocol** |  |  |
| Manufacturer of dPCR instrument and instrument model | Y | ABI 7500 Real-Time PCR System (Thermo Fisher Scientific). |
| Buffer/kit manufacturer with catalogue and lot number | Y | Platinum Quantitative PCR SuperMix-UDG with ROX reagent (reference nº 11743-500) for TaqMan amplification system or SYBR Select Master Mix (reference nº 4472908) for SYBR Green amplification system, both from Thermo Fisher Scientific. |
| Primer and probe concentration | Y | Primers and probes were added into the final PCR reaction volume at 1x concentration. |
| Pre-reaction volume and composition (incl. amount of template and if restriction enzyme added) | Y | For TaqMan amplification system the final reaction volume of 20μL was composed of: a master mix (10μL), RNase-free water (8μL), corresponding TaqMan probe (1μL), and the sample of study (1μL). For SYBR Green amplification system the final reaction volume of 20μL was composed of: a master mix (10μL), RNase-free water (7μL), corresponding amplification primers (1μL forward + 1μL reverse), and the sample of study (1μL). |
| Template treatment (initial heating or chemical denaturation) | Y | Initial heating is composed of 2 consecutive holding stages. First the temperature is raised from 25°C to 50°C within 2 minutes, and then the temperature is raised again up to 95°C for 10 minutes. |
| Polymerase identity and concentration, Mg++ and dNTP concentrations | Y | Platinum Quantitative PCR SuperMix-UDG with ROX reagent (reference nº 11743-500) and SYBR Select Master Mix (reference nº 4472908) both from Thermo Fisher Scientific. Mg++: 50 mM. dNTP concentration not available. |
| Complete thermocycling parameters | Y | Complete thermocycling parameters include the 2 consecutive initial heating steps describe above followed by 40 cycling stages. In each of these cycles the temperature is kept at 95°C for 15 seconds, and then it is dropped to 60°C for 1 minute. In the end the melt curve stage is performed with the following temperatures: 95°C for 15 seconds, 60°C for 1 minute, 95°C for 30 seconds, and 60°C for 15 seconds. |
| **8. Assay validation** |  |  |
| Details of optimization performed | Y | Commercial TaqMan probes are supplied with optimization checking by supplier. |
| Analytical specificity (vs. related sequences) and limit of blank (LOB) | Y | Assays amplify the intended target at least 10 Ct values earlier than the gene with the closest sequence homology (as provided by supplier). |
| Analytical sensitivity/LoD and how this was evaluated | Y | Sensitivity (number of template copies that an assay can detect above NTC background): Ct of a 10-copy sample (Ct ~35) less than the Ct of the NTC with a p-value <0.05 (as provided by supplier). |
| Testing for inhibitors (from biological matrix/extraction) | N | Not performed. |
| **9. Data analysis** |  |  |
| Description of dPCR experimental design | N | Not applicable. |
| Comprehensive details negative and positive of controls (whether applied for QC or for estimation of error) | Y | Intrinsic design of the experiments considers "BAT" samples as positive controls. Negative controls were samples without RT. |
| Partition classification method (thresholding) | Y | Randomized distribution in plates. |
| Examples of positive and negative experimental results (including fluorescence plots in supplemental material) | Y | Supplementary Figure 1 is attached showing fluorescent plots of *Ucp1* transcript measurements (positive) and negative controls (no RT added). |
| Description of technical replication | Y | Assays were performed in triplicate. |
| Repeatability (intra-experiment variation) | Y | < 0.3 % |
| Reproducibility (inter-experiment/user/lab etc. variation) | Y | < 1.8 % |
| Number of partitions measured (average and standard deviation) | N | Not applicable. |
| Partition volume | N | Not applicable. |
| Copies per partition (λ or equivalent ) (average and standard deviation) | N | Not applicable. |
| dPCR analysis program (source, version) | Y | 7500 Software Version 2.0.1. (Applied Biosystems). |
| Description of normalization method | Y | Normalization was performed on the basis of the 2(-DeltaDelta C(T)) method, using *Ppia* (TaqMan assays) or *Rps9* (SYBRoligos) as reference transcripts. |
| Statistical methods used for analysis | Y | Data have been expressed as mean ± standard error of the mean with respect to BAT under the control condition, which was adjusted to have a mean equal to 1. Putative outliers were detected and removed prior to statistical analyses using the Grubb's test, and the Shapiro-Wilk test was applied to establish the normality of datasets. A statistically significant difference between two groups was assessed by two-tailed unpaired Student's *t*-test. For datasets that did not follow a normal distribution, differences were evaluated using a two-tailed unpaired nonparametric Mann-Whitney's *u-*test. One-way analysis of variance (ANOVA) followed by Tukey Kramer's multiple comparisons post-hoc test was used to compare three or more groups. Statistical analyses were performed with GraphPad Prism 8 (GraphPad Software Inc.) and the programming environment R. In all cases, the statistical significance threshold was set at p<0,05. |
| Data transparency | Y | The raw data supporting the conclusions of this article will be made available by the authors, without undue reservation. |

**4 Supplementary Figure 1:** fluorescent plot exemplifying positive and negative experimental qRT-PCR results. The *Ucp1* amplification curves correspond to qRT-PCR assays from BAT-extracted RNA in the conditions depicted in Supplementary Table 2.

**
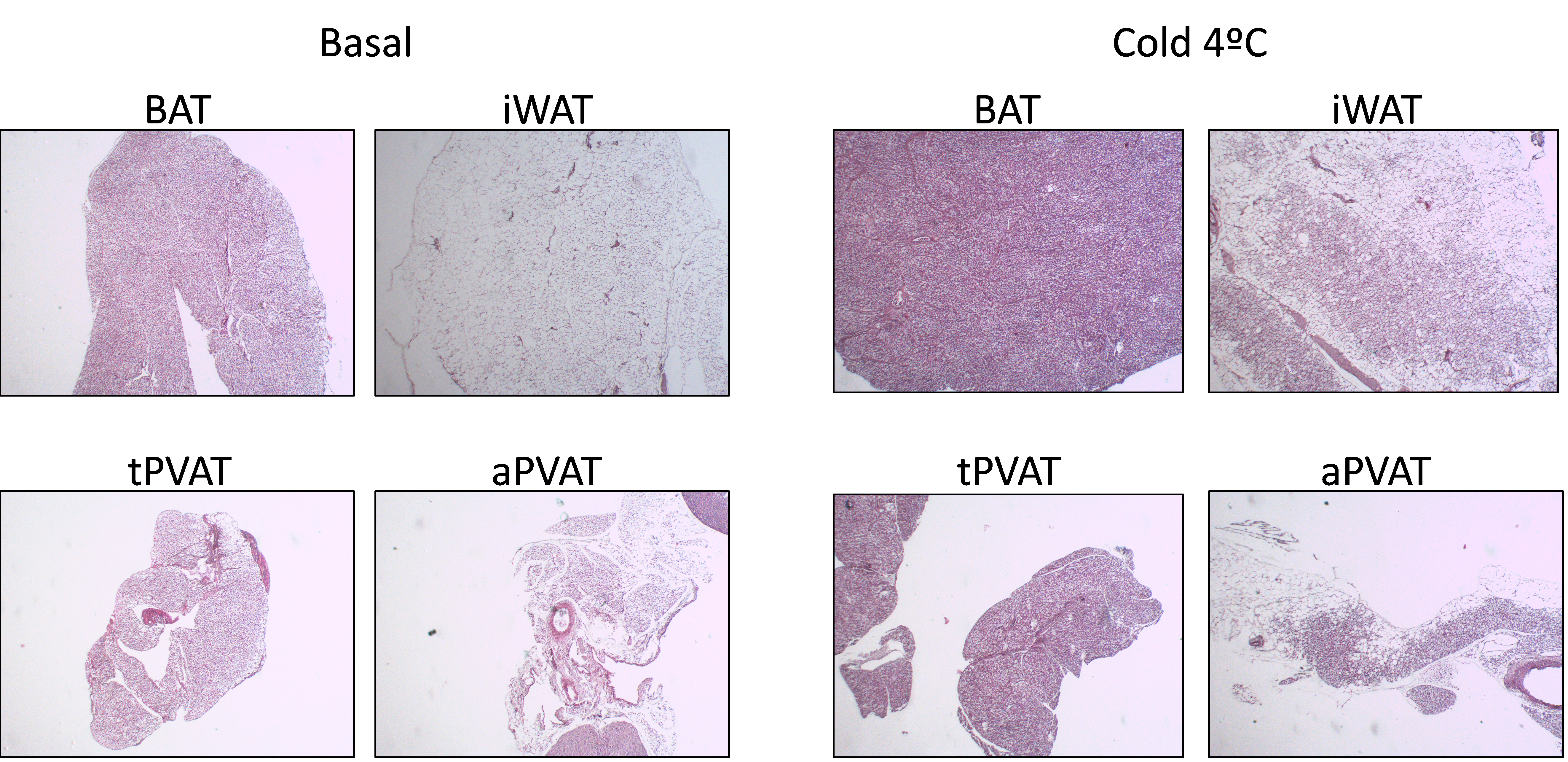
**

**5 Supplementary Figure 2:** representative optical microscopic pictures of hematoxylin-eosin stained sections of adipose tissue samples at low magnification (4X). On the left, histological appearance in the basal condition. On the right, adipose depots morphology after 1 week of cold exposure at 4°C.
